# Chickenpox at Ninety Four: A Case for Extending the Use of Varicella Vaccine in the UK

**DOI:** 10.1155/2010/561707

**Published:** 2010-06-09

**Authors:** Justin Bendig, Fiona Sindall

**Affiliations:** ^1^Department of Microbiology, Epsom and St. Helier University Hospitals NHS Trust, West Park Hospital, Horton Lane, Epsom, Surrey KT19 8PB, UK; ^2^Oxshott Medical Practice, Holtwood Road, Oxshott, Surrey KT22 0QL, UK

## Abstract

A case of chickenpox in a 94-year-old female is described. Serological tests for Varicella zoster virus (VZV) performed on early and late serum samples confirmed primary VZV infection. The patient recovered but seventeen days after presentation she developed a stroke from which she subsequently died. Chickenpox in older people is relatively rare—this case may be the oldest case of laboratory-proven chickenpox described—but it is a life-threatening illness. The varicella vaccine is licensed for use in immunocompetent persons of 12 months of age or older but in the UK is only offered to susceptible healthcare workers and close contacts of immunocompromised patients. In the US, the vaccine is recommended for all susceptible adolescents and adults. The same recommendation should be made in the UK.

## 1. Introduction

Chickenpox is considered a universal childhood infection in temperate parts of the world with >90% seropositivity by adolescence. If missed during childhood the next most likely time of exposure to VZV is probably when young adults have children of their own. A case of chickenpox is described in a 94-year-old female who had not had children. Chickenpox in older people is uncommon but life-threatening and could be prevented by wider use of varicella vaccine than is currently recommended in the UK. A case for this is made in light of relatively recent recommendations on the wider use of varicella vaccine in the US.

## 2. Case Report

A woman aged 94 years (and 50 weeks) presented to her general practitioner with a widespread rash of approximately 48-hour duration. She volunteered that she had never had chickenpox and that her husband had experienced ophthalmic zoster three weeks previously. On examination she did not appear unwell and was apyrexial. There was a vesicular rash on her face, limbs, and trunk consistent with chickenpox and no sign of localisation to a single dermatome. Famciclovir 250 mg, 8-hourly was started. On review the following day, the rash was very typical of chickenpox with some crusting of older lesions. She felt weak but remained apyrexial and had no chest symptoms or signs.

Serum collected on this second day of clinical review was positive for Varicella zoster virus (VZV) IgM antibody by enzyme immunoassay (EIA) (Trinity biotech) and negative for VZV IgG (EIA) consistent with early chickenpox infection. A second serum collected two days later showed the same VZV serology results, except that the IgM was even more reactive. A third follow-up serum collected a further fifteen days later revealed VZV IgG seroconversion to positive (strongly reactive) with the IgM remaining positive. The results confirmed primary VZV infection. A viral swab of the skin lesions was not collected.

Seven days after initial presentation the patient was recovering well and all skin lesions had crusted with earlier ones clearly showing signs of healing. A further ten days later, after making a full recovery, the patient suddenly developed a dense hemiplegia from which she died a month later. Computerised tomography confirmed an acute right hemisphere brain infarct associated with the middle cerebral artery. A postmortem was not performed.

## 3. Discussion

The absence of VZV-specific IgG antibody early in a chickenpox-like illness can be taken as evidence of primary VZV infection once subsequent seroconversion to IgG positive is demonstrated. It is well known that cell-mediated immunity to VZV wanes with old age, and that this is the mechanism of reactivated disease, but antibody levels can be expected to persist, even into the ninth and tenth decades of life in otherwise healthy older people [1]. This fact, together with the confident history volunteered by the patient described that she had never had chickenpox, satisfied us that this was a clear case of primary VZV infection.

The patient described was unlucky to be exposed to varicella virus for the first time at such a late age—the exposure almost certainly coming from her husband's ophthalmic zoster. A review of the literature reveals patients with chickenpox at 86 [2], 87 [3], and 88 years of age [4] with only the 87 year-old male surviving the illness. The patient described in this case report may be the oldest laboratory-proven case of chickenpox recorded.

A weakened cell-mediated immunity that we might expect in an elderly person would predispose to a more severe primary illness. Delayed or weakened virus-specific T-cell recognition has been shown to correlate with a more serious outcome in chickenpox [5]. Complications, such as pneumonitis, encephalitis, and secondary bacterial infection of skin lesions can also have more severe consequences in older people. To be effective, oral antivirals need to be started within 24 hours of onset of the chickenpox rash so it is unlikely that the famciclovir course (unlicensed in the UK if used for chickenpox) in the patient described was responsible for her uncomplicated recovery. It is interesting to speculate that the patient's hemiplegia was a briefly delayed complication of the chickenpox. Delayed contralateral hemiplegia is a well-described complication of ophthalmic zoster but delayed hemiplegia is also recognised as a rare complication of chickenpox in children [6]. Cerebrospinal fluid was not collected from the patient nor was postmortem tissue available for histological studies which might have provided evidence to support a causal association with the chickenpox. The patient also suffered atrial fibrillation and was not on warfarin so an embolic event associated with this condition might have been the sole cause of her stroke.

A live-attenuated varicella vaccine has been licensed for use in immunocompetent persons of 12 months of age or older for some years in the UK, but it is currently only offered to susceptible healthcare workers and household contacts of immunocompromised patients [7]. In the US the vaccine has been included in the routine childhood vaccination schedule since 1995 and it has been successful in reducing chickenpox morbidity and mortality, but it has only recently been formally recommended for all susceptible adolescents and adults [8]. Although no particular age group of adults is specified in the American recommendations it is generally assumed that the vaccine is not likely to be required by today's older adults, at least not to prevent chickenpox. Instead, studies on the use of varicella vaccine in the elderly have all been aimed at the prevention of Herpes zoster and this has required higher potency of Varicella zoster virus (OKA strain) than the 1350 plaque-forming units (PFU) contained in each 0.5 mL of “Varivax” vaccine (Merck). The recently licensed zoster vaccine, “Zostavax” (Merck) in the US has a potency of at least 14 times that of Varivax [9]. However, this does not mean that high potency is necessarily required to prevent chickenpox in the elderly. Trannoy et al. [10] achieved increased humoral and cell-mediated immunity in elderly subjects with a single dose of vaccine containing a potency of virus as low as 3200 PFU and did not try any lower potency. For adults, two doses of varicella vaccine are recommended (4–8 weeks apart); whether or not this will provide sufficient potency for older persons may never be tested in a clinical trial but we believe that susceptible elderly individuals—and we have shown they occur—could benefit. It would be useful to have case reports of individual elderly patients with their immunogenicity tested post vaccine; this would probably require, in addition to tests of cell-mediated immunity, more sensitive serological tests (such as the Time-Resolved Fluorescence Immunoassay [11]) than is routinely available in most medical virology laboratories as most commercial assays lack sensitivity to always detect vaccine-induced immunity to VZV [8].

In the UK, and other countries, the use of varicella vaccine in healthy children remains controversial; longer scrutiny of the cost benefits appears to be required. However, given the high risks associated with primary VZV infection in adulthood, it is hard to conceive why varicella vaccine cannot be offered to all susceptible adolescents and adults. If elderly persons are considered, in general, to be an unreliable group to question about a past history of chickenpox, either routinely or after exposure, at least the vaccination of younger adults may prevent even more serious disease when they are much older.

## References

[1] Burke BL, Steele RW, Beard OW, Wood JS, Cain TD, Marmer DJ (1982). Immune responses to varicella-zoster in the aged. *Archives of Internal Medicine*.

[2] Ho BCH, Tai DYH (2004). Severe adult chickenpox infection requiring intensive care. *Annals of the Academy of Medicine Singapore*.

[3] Savopoulos CG, Apostolopoulou MI, Hatzitolios AI (2006). Chickenpox in a geriatric patient. *Journal of the American Geriatrics Society*.

[4] Demissie A, Ayres RC (1989). Chickenpox in the elderly. *British Journal of Clinical Practice*.

[5] Arvin AM (1992). Cell-mediated immunity to varicella-zoster virus. *Journal of Infectious Diseases*.

[6] Ichiyama T, Houdou S, Kisa T, Ohno K, Takeshita K (1990). Varicella with delayed hemiplegia. *Pediatric Neurology*.

[7] Department of Health (2006). *Immunisation Against Infectious Disease*.

[8] Marin M, Güris D, Chaves SS, Schmid S, Seward JF (2007). Prevention of varicella: recommendations of the Advisory Committee on Immunization Practices (ACIP). *Morbidity and Mortality Weekly Report*.

[9] Oxman MN, Levin MJ, Johnson GR (2005). A vaccine to prevent herpes zoster and postherpetic neuralgia in older adults. *The New England Journal of Medicine*.

[10] Trannoy E, Berger R, Holländer G (2000). Vaccination of immunocompetent elderly subjects with a live attenuated Oka strain of varicella zoster virus: a randomized, controlled, dose-response trial. *Vaccine*.

[11] Maple PAC, Gray J, Breuer J, Kafatos G, Parker S, Brown D (2006). Performance of a time-resolved fluorescence immunoassay for measuring varicella-zoster virus immunoglobulin G levels in adults and comparison with commercial enzyme immunoassays and Merck glycoprotein enzyme immunoassay. *Clinical and Vaccine Immunology*.

